# Vitamin D supplementation in critically ill patients: a meta-analysis

**DOI:** 10.3389/fnut.2025.1505616

**Published:** 2025-04-30

**Authors:** Wen-He Zheng, Jia-Heng Shi, Da-Xing Yu, Hui-Bin Huang

**Affiliations:** ^1^Department of Critical Care Medicine, The Second People’s Hospital Affiliated to Fujian University of Traditional Chinese Medicine, Fuzhou, China; ^2^Department of Critical Care Medicine, Guang'anmen Hospital, China Academy of Chinese Medical Sciences, Beijing, China

**Keywords:** vitamin D, critical illness, mechanical ventilation, meta-analysis, mortality

## Abstract

**Background:**

Vitamin D is commonly used in clinical practice, while its clinical significance in critically ill patients remains controversial. Therefore, we aimed to perform a systemic review and meta-analysis to investigate the effect of vitamin D on this patient population.

**Methods:**

We searched for randomized controlled trials (RCTs) in PubMed, Embase, and the Cochrane Library databases from inception until August 15, 2024. Studies evaluating critically ill adult patients who received vitamin D compared to controls were included. The primary outcome was short-term mortality. We used the Cochrane risk of bias tool and GRADE system to evaluate the study quality and evidence. Secondary outcomes were changes in serum 25-hydroxyvitamin D levels, mechanical ventilation (MV) duration, and length of stay (LOS) in the ICU or hospital. We also conducted meta-regression, subgroup analyses, and trial sequential analysis (TSA) to explore the potential heterogeneity among the included trials.

**Results:**

Nineteen RCTs with 2,754 patients were eligible. Overall, vitamin D significantly increased serum 25-hydroxyvitamin D levels and significantly reduced the short-term mortality (risk ratio [RR] = 0.83; 95%CI, 0.70–0.98; *p* = 0.03, *I*^2^ = 13%), duration of MV (MD = −2.96 days; 95% CI, −5.39 to −0.52; *I*^2^ = 77%; *p* = 0.02) and ICU LOS (MD = −2.66 days; 95% CI, −4.04 to −1.29, *I*^2^ = 70%; *p* = 0.0001) but not hospital LOS (MD = −0.48 days; 95% CI, −2.37 to 1.40; *I*^2^ = 31%; *p* = 0.61). The meta-regression analysis revealed that the proportion of MV (MV%) accounted for the source of heterogeneity, and the subgroup analyses based on MV% suggested that the MV group was more likely to benefit from vitamin D applications than the partly MV group in all the predefined outcomes (all *p* values<0.05). TSA for short-term mortality suggested that more data is required to confirm our main conclusion.

**Conclusion:**

Vitamin D supplementation increased serum 25-hydroxyvitamin D levels and significantly benefited critically ill patients, especially those with MV.

**Systematic review registration:**

https://inplasy.com/inplasy-2022-10-0074/, INPLASY2022100074.

## Introduction

Vitamin D is a fat-soluble vitamin that regulates calcium-phosphorus levels in bone metabolism (1), as well as various body processes, including hormonal regulation, immunomodulatory, oxidative stress, cardiovascular, and muscular effects (2, 3). To achieve its bioactive hormone state, vitamin D must be hydroxylated in the liver and kidneys to form 25-hydroxyvitamin D (25-OHD) and 1,25-OHD (1). Previously published studies used a targeted value of 30 ng/mL 25-OHD levels to define vitamin D deficiency (VDD) according to the suggestion of Endocrine Society clinical practice guideline (4). However, in 2024, the up-dated guideline abandoned these recommendations because they did not find enough scientific confirmation (5). In contrast, the Institute of Medicine and European Food Safety Authority recommended 20 ng/mL (50 nmol/L) which has not changed (6, 7). In addition, other associations such as the International Osteoporosis Foundation and the American Geriatrics Society also use 30 ng/mL as a guideline goal (8, 9).

Nevertheless, VDD is common in critically ill patients (10). It is associated with severe complications (i.e., infection, acute liver failure, acute respiratory insufficiency), development of sepsis, prolonged mechanical ventilation (MV), and mortality (10, 11). In contrast, vitamin D supplementation has been shown to significantly improve the prognosis in mechanically ventilated patients (12). Therefore, vitamin D supplementation in critically ill patients may be essential and is recommended by some guidelines of clinical nutrition (13).

However, two large RCTs failed to show the prognostic value of vitamin D for critically ill patients (14, 15). Meta-analyses have produced conflicting results in recent years. Previous meta-analyses either included a limited number of trials or were contaminated with observational studies. This has led to non-robust results. Of note, three recently published meta-analyses reached inconsistent conclusions (16–18). Two of them did not support the survival benefits of vitamin D in critically ill patients (16, 18), but the third, published by Menger and colleagues, showed that vitamin D reduces mortality in this patient population (17). Some of these meta-analyses, which further explored factors like the route or dose of vitamin D administration, could not resolve the heterogeneity of their main results (16–18).

Interestingly, all of the authors’ perspectives were based on the assumption that supplemental vitamin D was associated with its increased concentration *in vivo* (16–18). However, they failed to provide relevant data for analyzing vitamin D concentrations. Furthermore, all three meta-analyses suggest that vitamin D significantly reduces the duration of MV, which is questionable (16–18). It also remains unclear whether the selected patient populations affected the results.

Considering the highly heterogeneous group of critically ill patients and variations in study design and implementation among the included studies, clarifying the optimal regimen for vitamin D is essential. Therefore, with the power of meta-analysis techniques, we aimed to include newly published RCTs to evaluate the effect of vitamin D on mortality and other important clinical outcomes in critically ill patients. We also explored the influences of vitamin D-associated factors with the help of meta-regression analysis and subgroup analyses.

## Method

The present study’s protocol has been registered on the International Platform of Registered Systematic Review and Meta-analysis Protocols database (Registration number: INPLASY2022100074). We conducted our study and adhered to the Preferred Reporting Items for Systematic Reviews and Meta-Analyses (PRISMA) statement (19) (Supplementary file 1).

### Eligibility criteria

Predefined criteria for eligible studies were as follows:

1. The studies should include critically ill adult patients;
2. The intervention group received vitamin D regardless of any regimen (i.e., dose, timing, and route administration);
3. The control group received a placebo or no drug or usual care;
4. The primary outcome was short-term mortality [defined as ICU or hospital or 28-day mortality, or mortality within 90 days after discharge from the hospital, the longest period was preferred (20)]. Secondary outcomes were serum changes between groups after the intervention, the length of stay (LOS) in the ICU or hospital, and the duration of MV;
5. RCTs.

We excluded studies conducted in pregnant women, reviews, case reports, case series, *post hoc* analyses, or studies that did not report any predefined outcomes.

### Search strategy

Two authors (W-HZ and J-HS) independently searched for eligible studies in the PubMed, Embase, and Cochrane Library databases before October 15, 2022, which was the last search. Details of the search strategy are summarized in Supplementary file 2. No language limitation was imposed. Grey literature^1^ was also searched. We also evaluated the reference lists of relevant studies and previous meta-analyses to ensure the inclusion of all potential studies. Discrepancies were identified and resolved by a third author (H-BH) with arbitration.

### Data extraction

The two authors (W-HZ and J-HS) extracted the data independently on the first author’s name, publication year, sample size, inclusion criteria, patient characteristics (age, gender, and disease severity), study quality, Vitamin D regimens, as well as predefined outcomes. Discrepancies were resolved by discussion. Authors of the included RCTs were contacted for missing or unclear information of primary outcomes if required.

### Quality assessment

The Cochrane risk of bias tool was used to assess the methodological quality of the individual RCTs (21). For each included trial, we assigned a risk of bias rating of “low,” “unclear,” or “high” for the following items: sequence generation, allocation concealment, blinding, incomplete outcome data, selective outcome reporting, and other biases. An individual trial’s overall risk of bias was classified as “low” (if the risk of bias was low in all domains), “moderate” (if the risk of bias was unclear in at least one domain, with no high risk of bias domains), or “high” (if the risk of bias was high in at least one domain). We also used the GRADE system to evaluate the quality of evidence. Disagreements were settled by discussion and consensus. When at least 10 trials were included in our meta-analysis, we evaluated publication bias by visually analyzing funnel plots and using Egger’s test.

### Statistical analysis

The results from all relevant studies were combined to estimate the pooled risk ratio (RR) and associated 95% confidence intervals (CI) for dichotomous outcomes. As to the continuous outcomes, we estimated mean differences (MD) and 95% CI as effective results. For studies that reported a median with an accompanying interquartile range (IQR) or range as the measure of treatment effect, we estimated the mean from the median and standard deviations (SD) from the IQR or range using the methods described in the previous studies before data analysis (22, 23). We used the *I*^2^ statistic to test the heterogeneity. An *I*^2^ < 50% was considered insignificant heterogeneity, and an *I*^2^ > 50% was regarded as substantial heterogeneity (24). We performed trial sequential analysis (TSA) for short-term mortality with the random-effect (DL) model to adjust the significance levels for sparse data and repetitive testing on accumulating data in the current meta-analysis (25). Thus, we defined the required information size for decreased mortality based on a risk of type I error of 5%, a risk of type 2 error of 20%, the control group outcome, and a relative risk reduction of 24.8 and 20% to calculate the required information size and the cumulative Z-curve’s eventual breach of relevant trial sequential monitoring boundaries. We performed all analyses using Review Manager, Version 5.3, TSA Viewer Version 0.9, and STATA Version 13.0 (College Station, TX, United States).

To test the robustness of the outcomes and explore the potential influence factors, we conducted sensitivity analyses to investigate the influence of a single study on the overall pooled estimate of each predefined outcome. We performed meta-regression based on several vitamin D-related clinical variables, including the proportion of patients with MV (MV%) (100% vs. mixed), vitamin D dose (≤300,000 UI vs. >300,000 UI), serum vitamin D level at baseline (VDD, defined as <20 ng/mL vs. <30 ng/mL vs. no threshold), design (single center vs. multicenter study), and route of vitamin D administration (enteral/oral vs. intravenous/intramuscular injection, IV/IM) to explore the possible source of heterogeneity among the included trials. Then the subgroup analyses were conducted according to the results of the meta-regression.

## Results

### Search results

**Figure 1:**
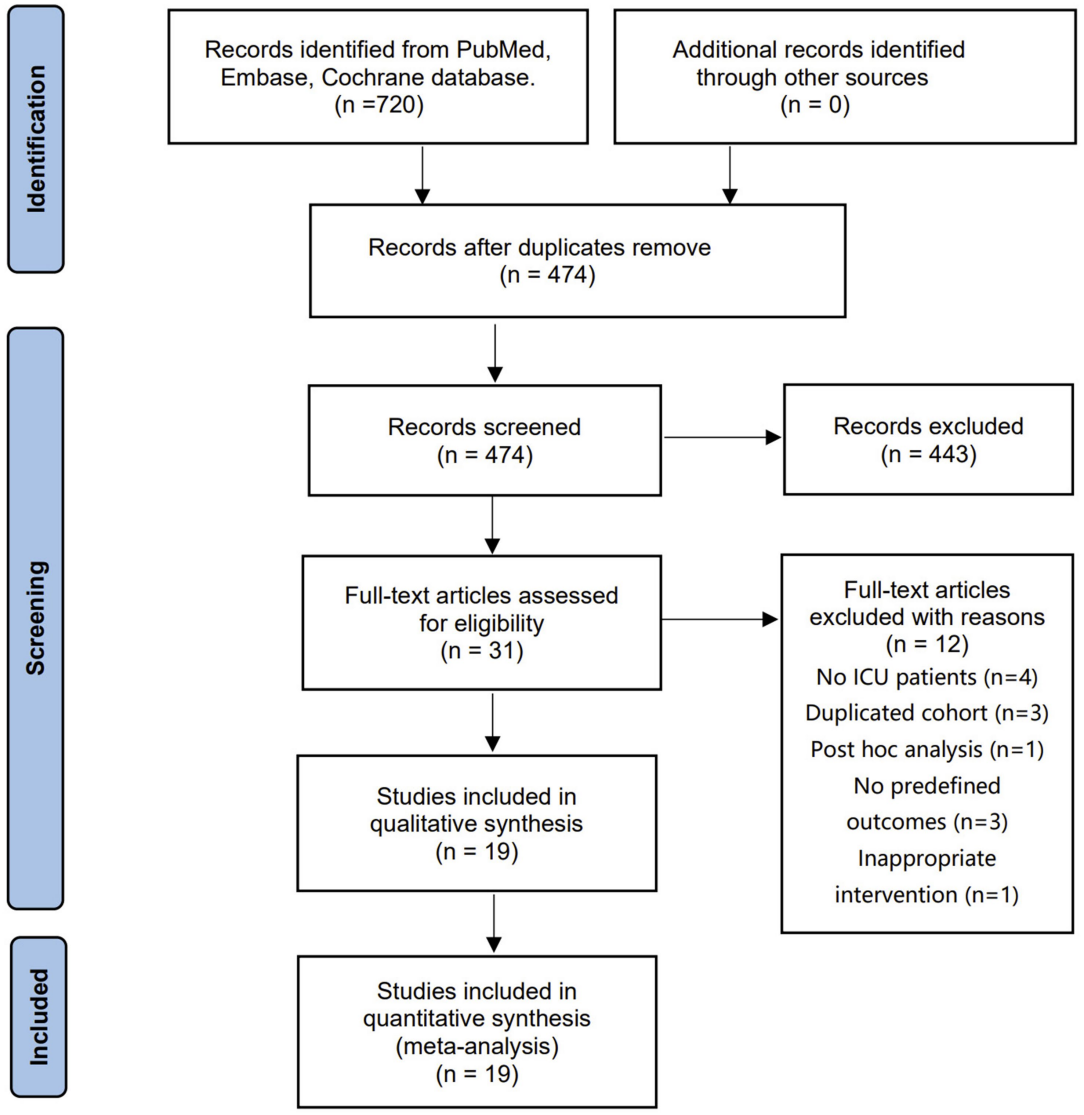
The selection process for studies included in the meta-analysis.

The electronic search retrieved 720 citations, 474 of which were selected from the de-duplicated results. After independently screening the titles and abstracts, we identified 31 relevant articles in full text for eligibility. Finally, 19 RCTs with 2,754 patients were included in the final analysis (14, 15, 26–42). Figure 1 shows a flowchart for selecting studies, and the studies needed for full review but not included in the current meta-analysis are summarized in Supplementary file 3.

### Studies characteristics

The main characteristics of the included studies are listed in Table 1. Eligible studies were published between 2011 and 2021 and were conducted in nine countries. 13 RCTs were conducted in multi-centers, while six were single-center studies (14, 15, 28, 33, 34, 36). All but one study (35) used the double-blinded design. Among the included trials, 12 focused on only patients with MV (27–29, 32–36, 38–42), and 10 provided clear VDD definition (defined as serum vitamin D < 20 ng/mL) (Supplementary file 4). Most of the included studies reported the details of the vitamin D regimen. Regarding the route of vitamin D administration, 11 RCTs used enteral/oral (14, 15, 26–28, 31, 35–39, 42), seven RCTs used IM/IV (30, 32–34, 39–41), while the remaining one evaluated both ways of vitamin D administration compared to usual care (29).

**Table 1:** Characteristics of the included studies.

Study	Country	Design	N	Age	VDD	MV%	Route	Vitamin D dose	ROB
Amrein 2011 (26)	Austria	SC, DB	25	61/64	100%	84%	Enteral	Single dose of 540,000 IU	L
Amrein 2014 (14)	Austria	MC, DB	492	64/65	100%	64%	Enteral	Loading dose of 540,000 IU; then 90,000 IU/month x 5	L
Leaf 2014 (32)	USA	SC, DB	67	68/58	Unlimited	70%	IV	Calcitriol 2 mcg	U
Ginde 2019 (15)	USA	MC, DB	1,078	57/55	100%	33%	Enteral	Single dose of 540,000 IU	L
Karsy 2019 (31)	USA	SC, DB	267	58/56	100%	Mixed	Enteral	Single dose of 540,000 IU	U
Quraishi 2015 (37)	USA	SC, DB	30	63/65	Unlimited	Mixed	Enteral	Single dose of 200,000 IU or 400,000 IU	L
Sharma 2021 (38)	India	SC, DB	35	36	100%	100%	Enteral	Single dose of 120,000 IU	U
Bhattacharyya 2021 (27)	India	SC, DB	126	42/44	100%	100%	Enteral	Single dose of 540,000 IU	L
Miroliaee 2017 (34)	Iran	MC, DB	46	67/59	100%	100%	IM	Single dose of 300,000 IU	U
Ding 2017 (41)	China	SC, DB	57	29/28	100%	100%	IM	Single dose of 300,000 IU	L
Miri 2019 (33)	Iran	MC, DB	40	64/72	100%	100%	IM	Single dose of 300,000 IU	U
Han 2016 (28)	USA	MC, DB	31	67/65	Unlimited	100%	Enteral	50,000 IU/day × 5 or 100,000 IU/day × 5	U
Hasanloei 2019 (29)	Iran	SC, DB	72	47/49	100%	100%	Enteral/IM	Single dose of 500,000 IU or 300,000 IU	U
Parekh 2018 (36)	UK	MC, DB	79	65/66	100%	100%	Enteral	Single dose of 300,000 IU	U
Sistanizad 2021 (39)	Iran	SC, DB	36	62/58	100%	100%	IM	Single dose of 300,000 IU	H
Yousefian 2019 (40)	Iran	SC, DB	99	70/67	100%	100%	IM	300,000 IU up to three doses per week	H
Naguib 2020 (35)	Egypt	SC, UB	89	44/43	Unlimited	100%	Enteral	Alfacalcidol 2 mg/d started two days before surgery	H
Ingels 2020 (30)	Belgium	SC, DB	24	58/52	100%	100%	IV	Loading 200 μg calcidiol; 15 μg/day × 10	U
Wang 2024 (42)	China	MC, DB	61	71/65	100%	83.6%	Enteral	Single dose of 75,000 IU × 8	U

DB, double blind; H, high risk; IM, intramuscular; IV, intravenous; L, low risk; MC, multi-centers; Mixed, only part patients received mechanical ventilation; ROB, risk of bias; SC, single center; U, unclear risk; VDD, vitamin D deficiency (defined as <30 ng/mL); UB, unblind.

### Risk of bias in studies

Six RCTs were considered at low risk of bias (14, 15, 26, 27, 37, 41), 10 were judged to be at moderate risk of bias (28–34, 36, 38, 42), and three trials were deemed at high risk of bias (35, 39, 41) (Supplementary file 5). Using GRADE methodology, we evaluated the evidence for short-term mortality and duration of MV to be low, whereas ICU LOS, hospital LOS, and changes in 25(OH)D concentrations were very low (Supplementary file 6). Assessment of publication bias using visually inspecting funnel plots showed no skewed distributions, suggesting no potential publication bias among the included trials (Supplementary file 7). We further investigated publication bias by conducting Egger tests; there was also no evidence of publication bias (Kendall’s tau = 0.0588, *p* = 0.7652).

### Serum 25-hydroxyvitamin D level

A total of 15 RCTs reported the serum 25(OH)D concentrations at baseline and after intervention (most at 3–7 days later, provided by each trial) (Supplementary file 8). Among them, 14 provided specific data that could be pooled (14, 15, 26–29, 31, 33, 36–39, 42). Overall, the changes in plasma 25(OH)D concentrations after intervention in the vitamin D group were more significant than in the control group (n = 1,519; MD = 12.89 ng/mL; 95% CI, 8.17 to 17.61; *I*^2^ = 96%; *p* < 0.00001). Similar results were also observed when only trials of vitamin D administered by enteral/oral (*p* < 0.00001), trials of vitamin D administered by IM/IV(*p* = 0.005), trials of high vitamin D dose (*p* < 0.00001), trials of low vitamin D dose (*p* < 0.00001), or Vit D administration in partly/100% MV cohort (*p* < 0.00001), were pooled, respectively. Details of results were summarized in Table 2.

**Table 2:** Comparison of the changes in serum 25-hydroxyvitamin levels between vitamin D and control in subgroup critically ill patients.

Subgroup	Included studies	N	Effect estimate; MD (95%CI)	*I*^2^	*P* value
All of the included studies	(14, 15, 26–31, 33, 36–39, 42)	1,519	12.89 [8.17, 17.61]	96	<0.00001
Vit D dose >300,000	(14, 15, 26–29, 31, 37, 42)	1,314	16.19 [9.65, 22.73]	97	<0.00001
Vit D Dose ≤300,000	(28–30, 33, 36–39)	272	8.73 [4.92, 12.55]	85	<0.00001
Vit D administration by IV/IM	(29, 33, 39)	142	6.57 [2.00, 11.14]	91	0.005
Vit D administration by EN/oral	(14, 15, 26–31, 36–38, 42)	1,449	14.49 [9.16, 19.81]	96	<0.00001
Vit D administration in partly MV cohort	(27–30, 33, 36, 38, 39, 42)	459	8.28 [4.73, 11.84]	86	<0.00001
Vit D administration in 100% MV cohort	(14, 15, 26, 31, 37)	1,060	19.15 [7.72, 30.59]	98	0.001

CI, confidence interval; N, enteral; IM, intramuscular; IV, intravenous; MD, mean difference; Vit D, vitamin D.

In addition, vitamin D administered by enteral/oral resulted more increased serum 25(OH)D concentrations than that by IV/IM (17.16 ng/mL vs. 5.72 ng/mL), while the high vitamin D dose administered had more increased serum concentrations than the low vitamin D dose (18.96 ng/mL vs. 9.96 ng/mL). However, the direct comparisons were unsuitable because of pooled results from different studies (Supplementary file 9).

### Primary outcome

**Figure 2:**
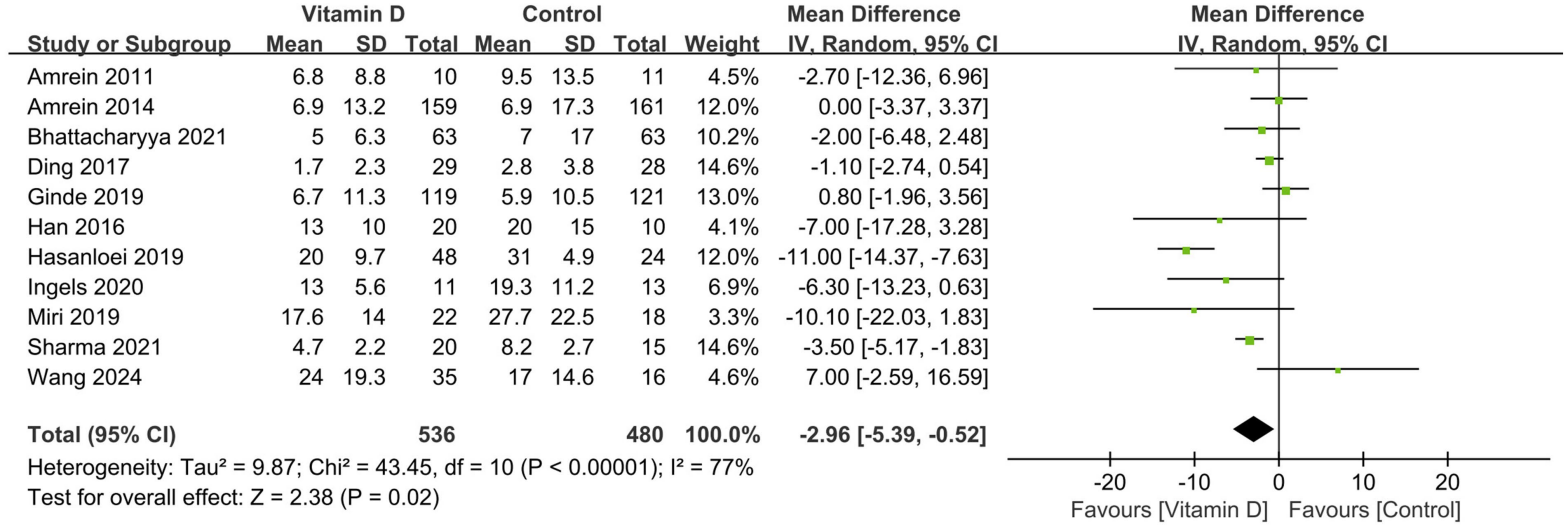
Forest plots of the effect of vitamin D supplementation on short-term mortality. Partly MV = only part of patients received mechanical ventilation. 100% MV = all patients received mechanical ventilation.

All 19 RCTs reported the outcome of short-term mortality. The pooled analysis showed that compared with the control group, vitamin D supplementation significantly reduced the risk of mortality (*n* = 2,664, RR = 0.83; 95% CI, 0.70 to 0.98; *I*^2^ = 13%; *p* = 0.03) (Figure 2) in critically ill patients. We proceeded to perform meta-regression analyses across predefined potential clinical factors. The results suggest that only MV% (*p* = 0.039) rather than vitamin D dose (*p* = 0.171), serum 25(OH)D level at baseline (0.134), single center or multicenter study design (0.958), and route of vitamin D delivery (*p* = 0.083) was the potential source of heterogeneity among the included trials (Supplementary file 10). Subsequently, we conducted subgroup analysis based on MV% and found that vitamin D significantly reduced the short-term mortality in the MV subgroup (0.65 [0.50, 0.84]; *p* = 0.0009) in comparison to that in the partial MV subgroup (0.98 [0.84, 1.15], *p* = 0.80), with no heterogeneity shown in both two subgroup findings (*I*^2^ = 0%) (Figure 2).

TSA for the results of short-term mortality was provided in Supplementary file 11. The cumulative z-curve crossed the conventional boundary (Z-statistic above 1.96) for benefit but did not cross the trial sequential monitoring boundary for benefit. Meanwhile, the number of patients included in TSA did not exceed the required information size of 5,001.

### Secondary outcomes

**Figure 3:**
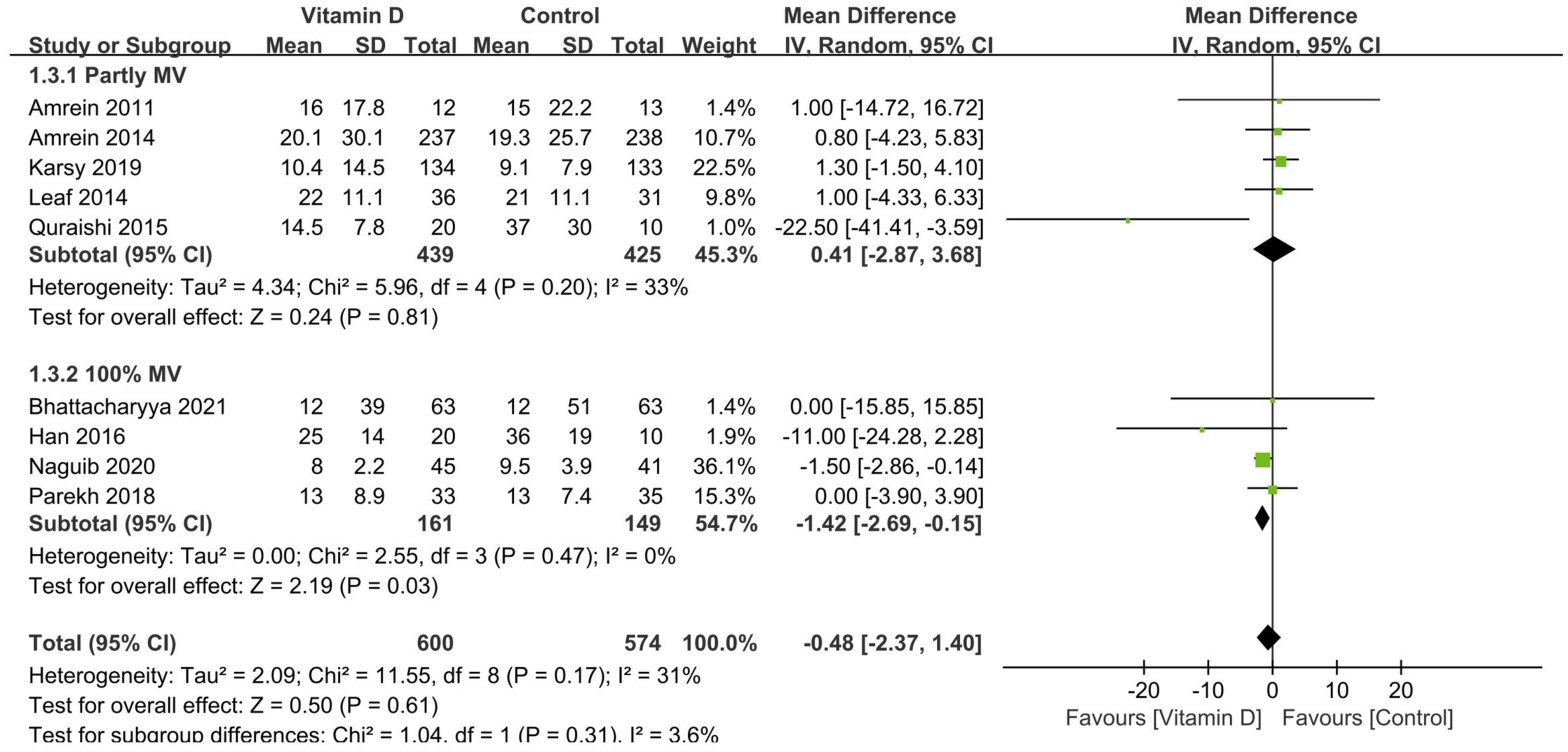
Forest plots of the effects of vitamin D supplementation on the duration of mechanical ventilation.

**Figure 4:**
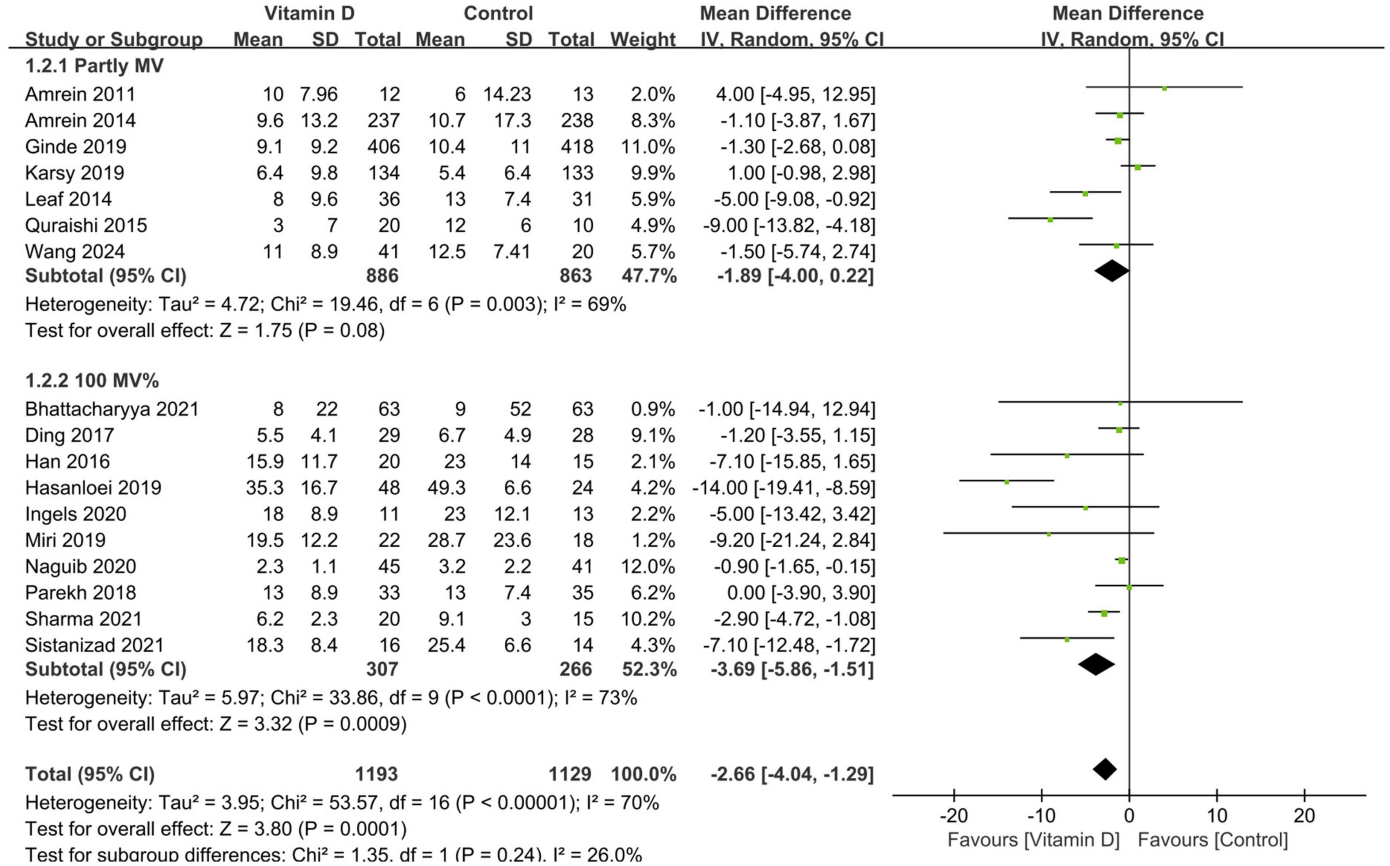
Forest plots of the effects of vitamin D supplementation on the length of stay in ICU. Partly MV = only part of patients received mechanical ventilation. 100% MV = all patients received mechanical ventilation.

**Figure 5:**
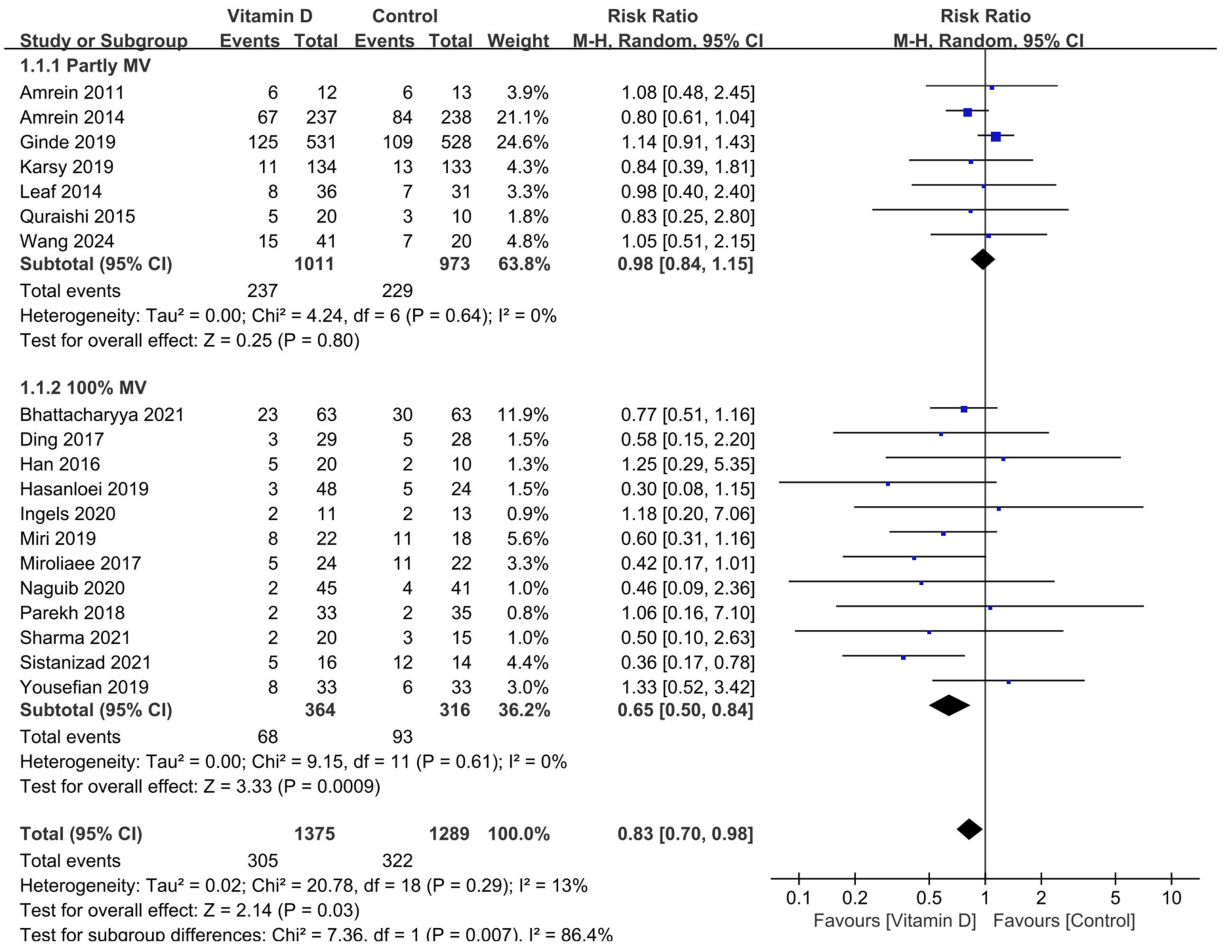
Forest plots of the effects of vitamin D supplementation on the length of stay in hospital. Partly MV = only part of patients received mechanical ventilation. 100 MV% = all patients received mechanical ventilation.

Compared with the control group, vitamin D supplementation significantly decreased the duration of MV (11 RCTs, n = 1,016, MD = −2.96 days; 95% CI, −5.39 to −0.52; *I*^2^ = 77%; *p* = 0.02) (Figure 3) (14, 15, 26–30, 33, 38, 41, 42) and the ICU LOS (17 RCTs, n = 2,322, MD = −2.66 days, 95% CI, −4.04 to −1.29, *I*^2^ = 70%, *p* = 0.0001) (Figure 4) (14, 15, 26–33, 35–39, 41, 42). Nine RCTs provided specific data on the outcome of hospital LOS and pooled results showed no significant difference between the groups (n = 1,174; MD = −0.48; 95% CI, −2.37 to 1.40; *I*^2^ = 31%; *p* = 0.61) (Figure 5) (14, 26–28, 31, 32, 35–37).

Similarly, all the subsequent subgroup analyses based on MV% suggested that vitamin D supplementation was associated with a significantly shorter length of stay in ICU (MD = −3.32 days; 95% CI, −5.40 to −1.25; *p* = 0.002) and hospital (MD = −1.42 days; 95% CI, −2.69 to −0.15; *p* = 0.03) in the MV subgroup rather than in the partly MV subgroup (Figures 4, 5).

## Discussion

This systematic review and meta-analysis suggested that vitamin D significantly reduced short-term mortality, duration of MV, and ICU LOS in ICU patients. In addition, patients with MV may benefit more from vitamin D administration.

### Possible explanations of the results of our research

Some mechanisms may explain our results. Vitamin D has been demonstrated to regulate skeletal muscle function through its active form, 1,25(OH)D, which is involved in muscle production, cell proliferation, differentiation, and regulation of protein synthesis (43). In animal studies, Foong et al. suggested that VDD causes airway hyperresponsiveness and increases airway smooth muscle mass in mice (44). Another study found that circulating 25(OH)D levels of 20 ng/mL caused smaller diaphragm muscle fiber diameter and a decreased ability to generate inspiratory force (45).

Compared with the adequate evidence that normal individuals benefit from vitamin D for skeletal muscle, the evidence in critically ill patients is limited. Some studies suggest that severe VDD is correlated with the severity of COPD (46). In one RCT focusing on ventilated patients, the authors found that high-dose vitamin D supplementation significantly increased hemoglobin (Pgroup*time = 0.01) and improved iron metabolism (47). The authors suggested that vitamin D increases haemoglobin concentration in critically ill adults by modulating iron modulators (47). Previous study has shown anemia is independently associated with extubation failure (48). Red blood cell transfusion in patients with severe COPD leads to a significant reduction of both the minute ventilation and work of breathing (49) and was more able to successfully wean these patients from mechanical ventilation (50). Yousefian et al. (40) showed that patients with vitamin D deficiency or insufficiency had more difficulty getting off the ventilator than patients with normal vitamin levels. Also, vitamin D supplementation significantly reduced the duration of MV in critically ill patients and, to some extent, reduced the occurrence of ventilator-related lung injury (12, 51, 52). In addition, several studies reported that VDD could cause muscle fiber atrophy and sarcopenia (53, 54).

### Our results in relation to previous reviews

Our results contradict the findings of the two most recent meta-analyses (16, 18). The authors reported that supplemental vitamin D did not significantly reduce the mortality of ICU patients compared with controls. However, the lack of adequate published literature inclusion (18), the selection of only high-dose vitamin D RCTs (16), and the deficiency of exploration of heterogeneity sources (16, 18) might contribute to the unexplained bias and heterogeneity among their included trials. Moreover, at least 44 and 47% of patients without MV were included in their mortality results, respectively. In contrast, our results are consistent with another new meta-analysis by Menger et al. (n = 16 RCTs) (17). And yet, the authors conducted a subgroup analysis based on the route of vitamin D administration and found IV/IM vitamin D (RR 0.59, 95% CI, 0.42–0.82) significantly reduced overall mortality compared with enteral/oral administration (RR 0.90, 95% CI, 0.71–1.15) (17). Thus, they believed IV/IM administration might have a more significant impact on mortality. Notably, the proportion of patients with MV was less than 50% in the enteral/oral subgroup, compared with 95% in the IV/IM subgroup. Thus, the greater effects on mortality reported by Menger et al. were more likely due to the more ventilated patients in the IM/IV group (95% vs. 50%) rather than the reduced mortality with IV/IM administration. In addition, compared with other RCTs in our meta-analysis, the VIOLET study (15), a recent large RCT, reported that early administration of high-dose enteral vitamin D did not significantly reduce the 90-day mortality or other clinical outcomes among critically ill. Similarly, only 33% of included patients received MV in that study.

To address these shortcomings, we performed a comprehensive search that included 19 RCTs of 2,754 patients, giving us more statistical power to examine our primary outcome. We further identified the heterogeneity by meta-regression analyses. Subgroup analysis based on MV% resolved the statistical heterogeneity among the included trials. Finally, all secondary outcomes showed that a subgroup of patients with MV could benefit more from vitamin D, lending credence from clinical practice to the robustness of our main result. In agreement, the aforementioned recent meta-analyses (16–18) consistently reported that the use of vitamin D significantly reduced the duration of MV, an outcome only for ventilated patients. This, yet again, validates our findings.

### Discussion of the literature included

Although our meta-regression analysis suggested that only MV% was the primary source of heterogeneity, several potential influences are still worth discussing, such as the route of administration, dose, and baseline vitamin D status when vitamin D supplements are administered. Theoretically, IM/IV vitamin D application may be more appropriate than enteral administration for critically ill patients for the commonly seen intestinal dysfunction in this patient population (55). Thus, patients are more likely to achieve serum 25(OH)D concentrations via IM/IV application than enteral administration. Surprisingly, our studies found that vitamin D via enteral/oral resulted in more significant increases in serum 25(OH)D concentrations than IV/IM (18 ng/mL vs. 4.1 ng/mL) than via IM/IV. This might be associated with the fact that most of these enteral/oral patients (78%) received high-dose vitamin D (≥500,000 IU), while all the IM/IV patients used vitamin D of not more than 300,000 IU. In addition, two included RCTs showed adequate gastrointestinal absorption by the enteral active form of vitamin D (32, 35). Leaf et al. found significantly increased plasma 1,25(OH)D levels in calcitriol-versus placebo-treated patients (75.7 vs. 16.9 pg./mL; *p* < 0.001) (32), whereas Naguib et al. reported that the enteral alfacalcidol change in serum 25 (OH)D was −9.2% (32.7%) in the control group, compared to 25.1% (36%) in the intervention group (35). Thus, our findings support enteral vitamin D with the current dose for critically ill patients. Considering that most serum 25(OH)D concentrations come from different studies, more data in the future are needed to confirm our findings.

The dosing regimen of vitamin D varied among the included trials, with more than 90% of patients receiving 300,000 IU or above. A recent meta-analysis has reported that high-dose vitamin D (≥300,000 IU) application could not significantly reduce mortality in critically ill patients (35). Our findings revealed that giving high-dose vitamin D does result in higher 25(OH)D concentrations (18.96 vs. 9.96 ng/mL). However, such high 25(OH)D concentrations did not translate into an improvement in mortality compared to low concentrations (RR 0.59, 95% CI, 0.94–0.80, vs. RR 0.57, 95% CI, 0.41–0.80). This finding appears to be explained in part by the difference in the MV% between the two groups, i.e., about 50% of patients with high 25(OH)D concentrations received MV compared to the low 25(OH)D group, in whom 95% of patients received MV. Thus, pursuing higher doses of vitamin D than currently available is unnecessary in future studies.

In the current study, we used <30 ng/mL vs. <20 ng/mL vs. no threshold limit for the subgroup by referring to other studies (17). So, our results do not clarify the efficacy evaluation of patients with severe VDD (<10 ng/mL). It is noteworthy that the two included large RCTs came to different conclusions regarding vitamin D supplementation in a subgroup of people with severe VDD (defined as ≤12 ng/mL) (14, 15). The authors of the VITdAL-ICU trial observed lower in-hospital mortality in these patients (*p* = 0.04) (14), whereas Ginde et al. did not find a difference in mortality in their VIOLET trial (15). These opposite results might relate to the fact that the VITdAL-ICU trial used a loading dose of vitamin D followed by a maintenance dose, while the VIOLET trial did not. Again, we observed that the VITdAL-ICU trial (14) had a higher proportion of ventilated patients than the Ginde et al. study (15) (64% vs. 33%). Fortunately, the ongoing multinational, multicenter VITDALIZE trial (56) evaluating the efficacy of high doses of vitamin D (loading a dose of 540,000 IU, followed by 4,000 IU daily for 90 days) in patients with severe VDD (≤12 ng/mL) will provide hope for exploring this critical issue.

### Study limitation

Firstly, most of the RCTs included in the current meta-analysis had a sample size of fewer than 200 patients, which may overestimate the effect of our findings. Second, despite our meta-regression as well as subgroup analyses, there was still significant heterogeneity. Specially, variations in several definitions of vitamin D regimens, such as supplemental timing, frequency, and maintenance dose, as well as differences in treatment techniques, including sun exposure, age, adiposity, and gut function might cause heterogeneity and further compromise the robustness of our results (52, 57). Thirdly, the current meta-analysis only evaluated a single nutrient supplementation without considering the impact of other nutritional support treatments, including nutritional risk, energy and protein regimen, feeding intolerance, etc. Finally, we only assessed several clinically controversial factors for heterogeneity sources and may have missed some potential influences that need to be explored in future research.

## Conclusion

In this updated meta-analysis, we demonstrate that vitamin D supplementation increased plasma 25(OH)D levels and significantly improved short-term mortality in critically ill patients with MV. In addition, ICU LOS and duration of MV were significantly reduced in this patient population. More well-designed RCTs are needed to validate our conclusions.

## Data Availability

The original contributions presented in the study are included in the article/Supplementary material, further inquiries can be directed to the corresponding author.

## Footnotes

^1^https://www.basesearch.net; https://scholar.google.com
