# Supplementary material for: Vitamin D supplementation in critically ill patients: a meta-analysis
Source: Front Nutr. 2025 Apr 30;12:1505616. doi: 10.3389/fnut.2025.1505616 (PMC12075268; doi:10.3389/fnut.2025.1505616)
Supplement: Supplementary file 1 [file Table_1.docx]

**Vitamin D supplementation in critically ill patients: An up-dated meta-analysis**

*Corresponding author: Hui-Bin Huang,

Email: [hhba02922@btch.edu.cn](mailto:hhba02922@btch.edu.cn).

**Additional files**

Additional file 1 PRISMA checklist……………………………………………………………………………………………………………………………………………………………………………………………….……………………………………..2

Additional file 2 Search Strategy………………………………………………………………………………………………………………………………………………………………………………………………………………….……………………..5

Additional file 3 Studies needed for full-reviewed but not included in the current meta-analysis …………………………………………………………………………………………………..………….……………………..8

Additional file 4 Definition of VDD described in the included studies………………………………………………………………………………………………………………………………………………………………………………..9

Additional file 5 Assessment of study quality …………….…………………………….…………………………………………………………………………………………………………………………..…………………………………....……11

Additional file 6 GRADE evaluation…………………………. …………….…………………………….………………………………………………………………………………………………………………………………………………....……….12

Additional file 7 Funnel plot of comparison: short-term mortality…..…………………………………………………………………………………………………………………………….………………………………………....………13

Additional file 8 Serum 25-hydroxyvitamin D level at baseline and after treatment and their changes in levels after treatment between the two groups……………………………………………....14

Additional file 9 Changes in Serum 25-hydroxyvitamin D level after treatment (ng/ml) in the subgroup patients………….…….………..……………………….………………………………………..……....……..15

Additional file 10 Meta-regression analyses in outcome of mortality among included studies based on predefined clinical factors…………………………………………………..….…….…….……....……..16

Additional file 11 Trial sequential analysis of short-term mortality………………………………………………………………………………………………………………………………………………………….…….…….…....……...18

**Additional File 1**

**PRISMA 2009 checklist**

| **Section/topic** | **#** | **Checklist item** | **Reported on page #** |
| --- | --- | --- | --- |
| **TITLE** | | |  |
| Title | 1 | Identify the report as a systematic review, meta-analysis, or both. | 1 |
| **ABSTRACT** | | |  |
| Structured summary | 2 | Provide a structured summary including, as applicable: background; objectives; data sources; study eligibility criteria, participants, and interventions; study appraisal and synthesis methods; results; limitations; conclusions and implications of key findings; systematic review registration number. | 3 |
| **INTRODUCTION** | | |  |
| Rationale | 3 | Describe the rationale for the review in the context of what is already known. | 5 |
| Objectives | 4 | Provide an explicit statement of questions being addressed with reference to participants, interventions, comparisons, outcomes, and study design (PICOS). | 6 |
| **METHODS** | | |  |
| Protocol and registration | 5 | Indicate if a review protocol exists, if and where it can be accessed (e.g., Web address), and, if available, provide registration information including registration number. | 7 |
| Eligibility criteria | 6 | Specify study characteristics (e.g., PICOS, length of follow-up) and report characteristics (e.g., years considered, language, publication status) used as criteria for eligibility, giving rationale. | 7 |
| Information sources | 7 | Describe all information sources (e.g., databases with dates of coverage, contact with study authors to identify additional studies) in the search and date last searched. | 7 |
| Search | 8 | Present full electronic search strategy for at least one database, including any limits used, such that it could be repeated. | 7 -8 and Additional File 2 |
| Study selection | 9 | State the process for selecting studies (i.e., screening, eligibility, included in systematic review, and, if applicable, included in the meta-analysis). | 7 |
| Data collection process | 10 | Describe method of data extraction from reports (e.g., piloted forms, independently, in duplicate) and any processes for obtaining and confirming data from investigators. | 8 |
| Data items | 11 | List and define all variables for which data were sought (e.g., PICOS, funding sources) and any assumptions and simplifications made. | 8 |
| Risk of bias in individual studies | 12 | Describe methods used for assessing risk of bias of individual studies (including specification of whether this was done at the study or outcome level), and how this information is to be used in any data synthesis. | 8 |
| Summary measures | 13 | State the principal summary measures (e.g., risk ratio, difference in means). | 8 |
| Synthesis of results | 14 | Describe the methods of handling data and combining results of studies, if done, including measures of consistency (e.g., I^2^) for each meta-analysis. | 9 |

| Risk of bias across studies | 15 | Specify any assessment of risk of bias that may affect the cumulative evidence (e.g., publication bias, selective reporting within studies). | 8 |
| --- | --- | --- | --- |
| Additional analyses | 16 | Describe methods of additional analyses (e.g., sensitivity or subgroup analyses, meta-regression), if done, indicating which were pre-specified. | 9-10 |
| **RESULTS** | | |  |
| Study selection | 17 | Give numbers of studies screened, assessed for eligibility, and included in the review, with reasons for exclusions at each stage, ideally with a flow diagram. | 11 and Figure 1, Additional File 3 |
| Study characteristics | 18 | For each study, present characteristics for which data were extracted (e.g., study size, PICOS, follow-up period) and provide the citations. | 11  Table 1, |
| Risk of bias within studies | 19 | Present data on risk of bias of each study and, if available, any outcome level assessment (see item 12). | 12 |
| Results of individual studies | 20 | For all outcomes considered (benefits or harms), present, for each study: (a) simple summary data for each intervention group (b) effect estimates and confidence intervals, ideally with a forest plot. | 12 |
| Synthesis of results | 21 | Present results of each meta-analysis done, including confidence intervals and measures of consistency. | 12-14 |
| Risk of bias across studies | 22 | Present results of any assessment of risk of bias across studies (see Item 15). | Additional File 4 |
| Additional analysis | 23 | Give results of additional analyses, if done (e.g., sensitivity or subgroup analyses, meta-regression [see Item 16]). | 10-11  Additional File 5-6 |
| **DISCUSSION** | | |  |
| Summary of evidence | 24 | Summarize the main findings including the strength of evidence for each main outcome; consider their relevance to key groups (e.g., healthcare providers, users, and policy makers). | 15 |
| Limitations | 25 | Discuss limitations at study and outcome level (e.g., risk of bias), and at review-level (e.g., incomplete retrieval of identified research, reporting bias). | 19-20 |
| Conclusions | 26 | Provide a general interpretation of the results in the context of other evidence, and implications for future research. | 21 |
| **FUNDING** | | |  |
| Funding | 27 | Describe sources of funding for the systematic review and other support (e.g., supply of data); role of funders for the systematic review. | 22 |

**Additional File 2**

**Search strategy**

Database: PubMed, Embase, Cochrane library (Search completed 15th August 2024)

--------------------------------------------------------------------------------------------------------------------------------------------------------------------------------------------------------

**PubMed**

#5

Search: ((((((((((((((((Infants[Title/Abstract]) OR (Newborn[Title/Abstract])) OR (Newborn[Title/Abstract])) OR (Infant[Title/Abstract])) OR (Newborn[Title/Abstract])) OR (Infants[Title/Abstract])) OR (Newborns[Title/Abstract])) OR (Newborn[Title/Abstract])) OR (Neonate[Title/Abstract])) OR (Neonates[Title/Abstract])) OR ("Infant, Newborn"[Mesh])) OR ((child[Title/Abstract]) OR (children[Title/Abstract]))) OR ("Pediatrics"[Mesh])) OR ("Child"[Mesh])) AND (((((critical care[Title/Abstract]) OR (critically ill[Title/Abstract])) OR (intensive care[Title/Abstract])) OR ("Critical Care"[Mesh])) OR ((((sepsis[Title/Abstract]) OR (septic shock[Title/Abstract])) OR ("Sepsis"[Mesh])) OR ("Shock, Septic"[Mesh])))) AND (((((("Cholecalciferol"[Mesh]) OR ("Vitamin D"[Mesh])) OR ((((Vitamin D[Title/Abstract]) OR (Calciol[Title/Abstract])) OR (Vitamin D 3[Title/Abstract])) OR (Cholecalciferols[Title/Abstract]))) OR ("Calcitriol"[Mesh])) OR ((1, 25-(OH)2D3[Title/Abstract]) OR (1,25-Dihydroxycholecalciferol[Title/Abstract]))))) AND ((randomized controlled trial [pt] OR controlled clinical trial [pt] OR randomized [tiab] OR placebo [tiab] OR clinical trials as topic [mesh: noexp] OR randomly [tiab] OR trial [ti]) NOT (animals [mh] NOT humans [mh])) Sort by: Most Recent

#4

Search: (((((((((((((((Infants[Title/Abstract]) OR (Newborn[Title/Abstract])) OR (Newborn[Title/Abstract])) OR (Infant[Title/Abstract])) OR (Newborn[Title/Abstract])) OR (Infants[Title/Abstract])) OR (Newborns[Title/Abstract])) OR (Newborn[Title/Abstract])) OR (Neonate[Title/Abstract])) OR (Neonates[Title/Abstract])) OR ("Infant, Newborn"[Mesh])) OR ((child[Title/Abstract]) OR (children[Title/Abstract]))) OR ("Pediatrics"[Mesh])) OR ("Child"[Mesh])) AND (((((critical care[Title/Abstract]) OR (critically ill[Title/Abstract])) OR (intensive care[Title/Abstract])) OR ("Critical Care"[Mesh])) OR ((((sepsis[Title/Abstract]) OR (septic shock[Title/Abstract])) OR ("Sepsis"[Mesh])) OR ("Shock, Septic"[Mesh])))) AND (((((("Cholecalciferol"[Mesh]) OR ("Vitamin D"[Mesh])) OR ((((Vitamin D[Title/Abstract]) OR (Calciol[Title/Abstract])) OR (Vitamin D 3[Title/Abstract])) OR (Cholecalciferols[Title/Abstract]))) OR ("Calcitriol"[Mesh])) OR ((1, 25-(OH)2D3[Title/Abstract]) OR (1,25-Dihydroxycholecalciferol[Title/Abstract])))) Sort by: Most Recent

#3

Search: ((((("Cholecalciferol"[Mesh]) OR ("Vitamin D"[Mesh])) OR ((((Vitamin D[Title/Abstract]) OR (Calciol[Title/Abstract])) OR (Vitamin D 3[Title/Abstract])) OR (Cholecalciferols[Title/Abstract]))) OR ("Calcitriol"[Mesh])) OR ((1, 25-(OH)2D3[Title/Abstract]) OR (1,25-Dihydroxycholecalciferol[Title/Abstract]))) Sort by: Most Recent

#2

Search: ((((critical care[Title/Abstract]) OR (critically ill[Title/Abstract])) OR (intensive care[Title/Abstract])) OR ("Critical Care"[Mesh])) OR ((((sepsis[Title/Abstract]) OR (septic shock[Title/Abstract])) OR ("Sepsis"[Mesh])) OR ("Shock, Septic"[Mesh])) Sort by: Most Recent

#1

Search: (((((((((((((Infants[Title/Abstract]) OR (Newborn[Title/Abstract])) OR (Newborn[Title/Abstract])) OR (Infant[Title/Abstract])) OR (Newborn[Title/Abstract])) OR (Infants[Title/Abstract])) OR (Newborns[Title/Abstract])) OR (Newborn[Title/Abstract])) OR (Neonate[Title/Abstract])) OR (Neonates[Title/Abstract])) OR ("Infant, Newborn"[Mesh])) OR ((child[Title/Abstract]) OR (children[Title/Abstract]))) OR ("Pediatrics"[Mesh])) OR ("Child"[Mesh]) Sort by: Most Recent

--------------------------------------------------------------------------------------------------------------------------------------------------------------------------------------------------------

**Embase**

#16. #13 AND #14 AND #15

#15. 'clinical trial'/exp OR 'randomization'/exp OR 'single blind procedure'/exp OR 'double blind procedure'/exp OR 'randomized controlled trial'/exp OR 'crossover procedure'/exp OR 'placebo'/exp OR 'prospective studies'/exp OR ('randomi?ed controlled' NEXT/1 trial*) OR rct OR 'randomly allocated' OR 'allocated randomly' OR 'random allocation' OR (allocated NEAR/2 random) OR (single NEXT/1 blind*) OR (double NEXT/1 blind*) OR ((treble OR triple) NEAR/1 blind*) OR placebo*

#14. #5 OR #6 OR #7 OR #8 OR #9 OR #10 OR #11 OR #12

#13. #1 OR #2 OR #3 OR #4

#12. 'colecalciferol'/exp

#11. 'cholecalciferols':ab,ti AND ([embase]/lim OR [medline]/lim)

#10. 'calciol':ab,ti AND ([embase]/lim OR [medline]/lim)

#9. '1,25-dihydroxycholecalciferol':ab,ti AND ([embase]/lim OR [medline]/lim)

#8. '1, 25-(oh)2d3':ab,ti AND ([embase]/lim OR[medline]/lim)

#7. 'vitamin d3':ab,ti AND ([embase]/lim OR [medline]/lim)

#6. 'vitamin d':ab,ti AND ([embase]/lim OR[medline]/lim)

#5. 'vitamin d'/exp

#4. 'intensive care'/exp

#3. 'intensive care':ab,ti AND ([embase]/lim OR [medline]/lim)

#2. 'critically ill':ab,ti AND ([embase]/lim OR [medline]/lim)

#1. 'critical care':ab,ti AND ([embase]/lim OR [medline]/lim)

------------------------------------------------------------------------------------------------------------------------------------------------------------------------------------------------------

**Cochrane library**

#1 MeSH descriptor: [vitamin d] explode all trees

#2 ("vitamin d "):ti,ab,kw (Word variations have been searched)

#3 (cholecalciferols):ti,ab,kw (Word variations have been searched)

#4 (1,25-dihydroxycholecalciferol):ti,ab,kw (Word variations have been searched)

#5 (colecalciferol):ti,ab,kw (Word variations have been searched)

#6 (vitamin d3):ti,ab,kw (Word variations have been searched)

#7 (1, 25-(oh)2d3):ti,ab,kw (Word variations have been searched)

#8 #1 OR #2 OR #3 OR #4 OR #5 OR #6 OR #7

#9 (intensive care):ti,ab,kw (Word variations have been searched)

#10 (critical care):ti,ab,kw (Word variations have been searched)

#11 (respiratory distress syndrome):ti,ab,kw (Word variations have been searched)

#12 (sepsis):ti,ab,kw (Word variations have been searched)

#13 (shock):ti,ab,kw (Word variations have been searched)

#14 (systemic inflammatory response syndrome):ti,ab,kw (Word variations have been searched)

#15 (multiple organ failure):ti,ab,kw (Word variations have been searched)

#16 (respiratory insufficiency):ti,ab,kw (Word variations have been searched)

#17 (pulmonary ventilation):ti,ab,kw (Word variations have been searched)

#18 (burn):ti,ab,kw (Word variations have been searched)

#19 (critically ill):ti,ab,kw (Word variations have been searched)

#20 MeSH descriptor: [Critical Care] explode all trees

#21 MeSH descriptor: [Critical Illness] explode all trees

#22 #11 OR #12 OR #13 OR #14 OR #15 OR #16 OR #17#1 OR #18 OR #19 OR #20 OR #21

#23 (randomized controlled trial):pt (Word variations have been searched)

#24 #8 AND #22 AND #23

**Additional File 3**

**Table. Studies needed for full-reviewed but not included in the current meta-analysis (n=9 trials)**

| No | Study | Reason of exclusion |
| --- | --- | --- |
| 1 | Alizadeh N, Khalili H, Mohammadi M, et al. Effect of vitamin D on stress-induced hyperglycaemia and insulin resistance in critically ill patients. Int J Clin Pract. 2016 May;70(5):396-405. | No predefined outcomes |
| 2 | Han JE, Alvarez JA, Jones JL, Tangpricha V, Brown MA, Hao L, et al. Impact of high-dose vitamin D3 on plasma free 25-hydroxyvitamin D concentrations and antimicrobial peptides in critically ill mechanically ventilated adults. Nutrition. 2017 Jun;38:102-108. | Cohort duplicated from Han 2016 |
| 3 | Martucci G, McNally D, Parekh D, et al. Trying to identify who may benefit most from future vitamin D intervention trials: a post hoc analysis from the VITDAL-ICU study excluding the early deaths. Crit Care. 2019 Jun 4;23(1):200. | Post hoc analysis the VITDAL-ICU study without additional data |
| 4 | Smith EM, Jones JL, Han JE, et al. High-Dose Vitamin D3 Administration Is Associated with Increases in Hemoglobin Concentrations in Mechanically Ventilated Critically Ill Adults: A Pilot Double-Blind, Randomized, Placebo-Controlled Trial. JPEN J Parenter Enteral Nutr. 2018 Jan;42(1):87-94. | Data duplicated from Han 2016 |
| 5 | Smith EM, Jones JL, Han JE et al. High-Dose Vitamin D3 Administration Is Associated with Increases in Hemoglobin Concentrations in Mechanically Ventilated Critically Ill Adults: A Pilot Double-Blind, Randomized, Placebo-Controlled Trial. JPEN J Parenter Enteral Nutr. 2018 Jan;42(1):87-94. | Data duplicated from Peng 2004 |
| 6 | Talasaz AH, Salehiomran A, Heidary Z, et al. The effects of vitamin D supplementation on postoperative atrial fibrillation after coronary artery bypass grafting in patients with vitamin D deficiency. J Card Surg. 2022 Jul;37(7):2219-2224. | Inappropriate intervention and control |
| 7 | Cannata-Andía JB, Díaz-Sottolano A, Fernández P, et al. A single-oral bolus of 100,000 IU of cholecalciferol at hospital admission did not improve outcomes in the COVID-19 disease: the COVID-VIT-D-a randomised multicentre international clinical trial. BMC Med. 2022 Feb 18;20(1):83. | Report without ICU patients |
| 8 | Maghbooli Z, Sahraian MA, Jamalimoghadamsiahkali S, et al. Treatment With 25-Hydroxyvitamin D3 (Calcifediol) Is Associated With a Reduction in the Blood Neutrophil-to-Lymphocyte Ratio Marker of Disease Severity in Hospitalized Patients With COVID-19: A Pilot Multicenter, Randomized, Placebo-Controlled, Double-Blinded Clinical Trial. Endocr Pract. 2021 Dec;27(12):1242-1251. | Report without ICU patients |
| 9 | Murai IH, Fernandes AL, Antonangelo L, et al. Effect of a Single High-Dose Vitamin D3 on the Length of Hospital Stay of Severely 25-Hydroxyvitamin D-Deficient Patients with COVID-19. Clinics (Sao Paulo). 2021 Nov 26;76:e3549. | Report without ICU patients |
| 10 | Slow S, Epton M, Storer M, Thiessen R, Lim S, Wong J, et al. Effect of adjunctive single high-dose vitamin D3 on outcome of community-acquired pneumonia in hospitalised adults: The VIDCAPS randomised controlled trial. Sci Rep. 2018 Sep 14;8(1):13829. | Report without ICU patients |

**Additional File 4**

**Definition of VDD described in the included studies.**

| Study | Cut-off of VDD described in the included studies |
| --- | --- |
| Amrein 2011 | “Twenty-five patients (mean age 62 ± 16yrs) with vitamin D deficiency [25-hydroxyvitamin D (25(OH)D) **≤20 ng/ml**] and an expected stay in the ICU >48 hours were included and randomly received…” |
| Amrein 2014 | “…a medical and surgical population of 492 critically ill adult white patients with vitamin D deficiency (**≤20ng/mL**) assigned to receive either vitaminD3 (n = 249) or a placebo (n = 243).” |
| Han 2016 | “Several intensive care unit studies have demonstrated an association between vitamin D deficiency [25hydroxyvitamin D (25(OH)D) **< 20 ng/mL**] and increased hospital length of stay (LOS), readmission rate,  sepsis and mortality. |
| Ginde 2019 | “Of these patients, 1078 had baseline vitamin D deficiency (25-hydroxyvitamin D level, <**20 ng per milliliter** [50 nmol per liter]) confirmed by subsequent testing and were included in the primary analysis population.” |
| Karsy 2019 | “…and vitamin D deficiency, specifically measured by 25-hydroxyvitamin  D (defined as **≤ 20 ng/ml**).” |
| Miroliaee 2017 | “Vitamin D level in subjects were divided into 4 categories: <10 ng/mL = severely deficient, **10-20 ng/mL = deficient**, 20-30 ng/mL = insufficient,  and >30 ng/mL as normal.” |
| Yousefian 2019 | “Those included in group A (33 patients) had the vitamin D level of higher than **20 ng/ml** and those in the other group (66 patients) had the vitamin D level of **lower than 20 ng/ml** who themselves were further divided into the two groups of B and C randomly (each consisting of 33 patients).” |
| Naguib 2020 | “The findings from the present study show that vitamin D deficiency, defined as a serum 25(OH)D level of **<20 ng/mL,** was highly prevalent in critically ill patients…” |
| Ingels2020 | “Vitamin D deficiency is usually defined as a serum 25OHD concentration below **20 ng/ml** [3, 4]. Vitamin D deficiency has been associated with increased all-cause mortality [5, 6] and with poor outcome of several immune-related disorders [2, 7].” |
| Wang 2024 | “Patients who were 20 years or older; had serum vitamin D levels of  **less than 20 ng/mL**…” |
| Leaf 2014 | **No report.** |
| Quraishi 2015 | **No report.**  “..median baseline 25OHD level was 17 (IQR 13-22) ng/mL.” |
| Sharma 2021 | **No report.**  The serum VIT level was **18.30 (IQR 14.50–22.95**) ng/mL and **15.15 (IQR 11.80–26.90)** ng/mL in the case group) and control group. |
| Bhattacharyya 2021 | **No report.**  “Unlike the previous studies, we did not screen our patients for a vitamin D level and enrolled patients into the study irrespective of their baseline vitamin D levels.” |
| Parekh 2018 | **No reported**.  The serum VIT level was **46.4 (IQR 37.5–71.5) nmol/L** and **47.4 (IQR 34.5–68.1) nmol/L** in the intervention group and control group.  PS: ng/ml×3.12 = nmol/L |
| Sistanizad 2021 | **No reported**.  The serum VIT D level was (7.21 ± 2.31) ng/mL and (5.24 ± 0.71) ng/mL in the intervention group (n = 16) and control group (n = 14). |
| Ding 2017 | “…the sepsis patients with deficiency [25(OH)D3 **20-30 μg/L**] or insufficiency [25(OH)D3 < 20 μg/L] of vitamin D were divided into D3  treatment group (supplemented 300 kU vitamin D3) and placebo group (injected 1 mL physiological saline).” |
| Miri 2019 | VIT D (ng/dL) Patients with vitamin D deficiency (**<30 ng/dL**) |
| Hasanloei 2019 | “From 80 patients with traumatic injury and vitamin D deficiency randomly allocated in 3 groups…”. “All of the subjects were vitamin D deficient in plasma 25(OH)D levels between **10 and 30 ng/mL**” |

**Additional File 5**

**Assessment of study quality**


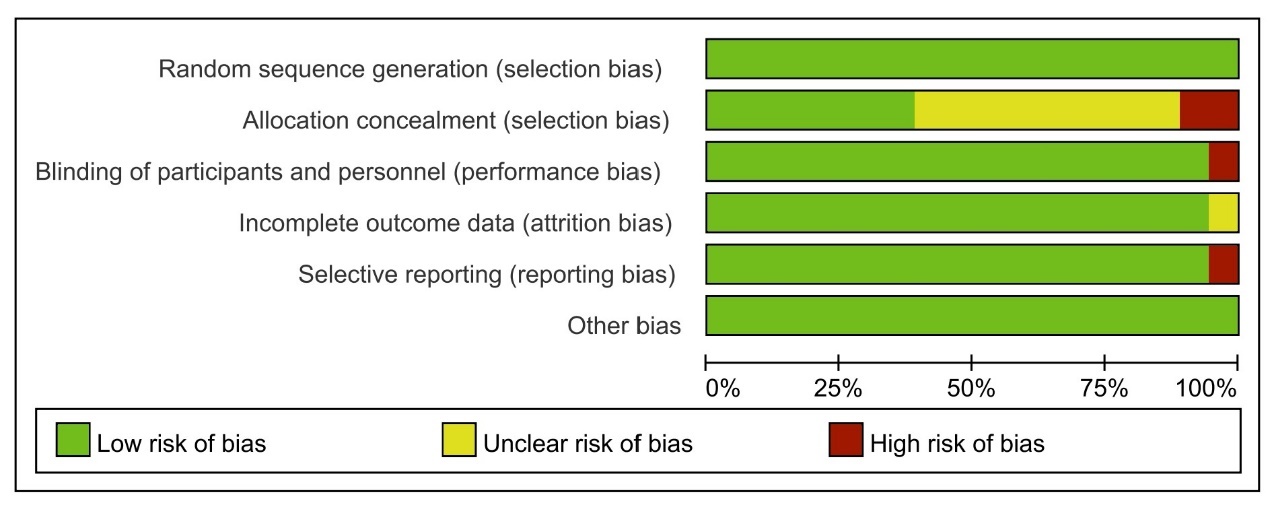


**Figure S1. Risk of bias graph: review authors' judgements about each risk of bias item presented as percentages across all included studies.**


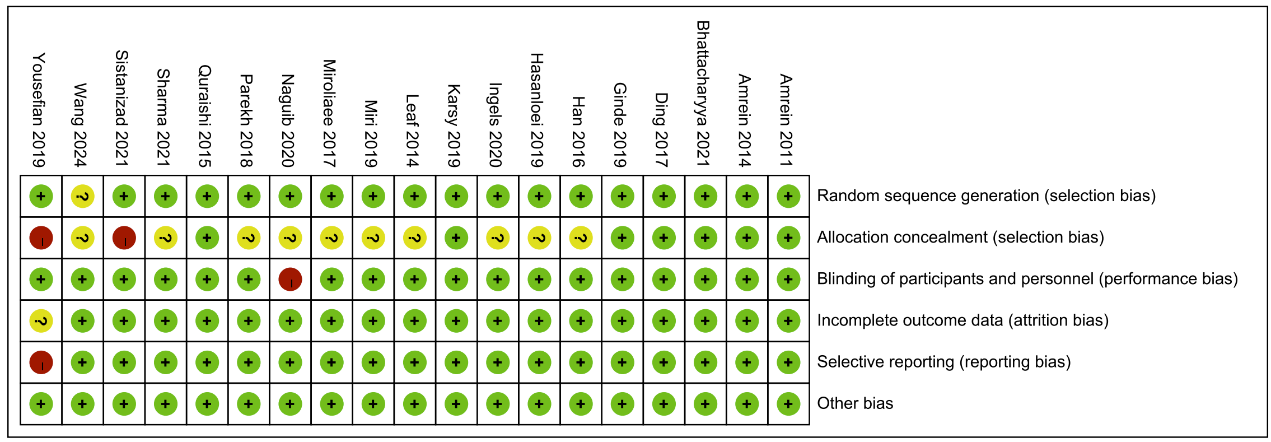


**Figure S2. Risk of bias summary: review authors' judgements about each risk of bias item for each included study.**

**Additional File 6: GRADE** **profile.**


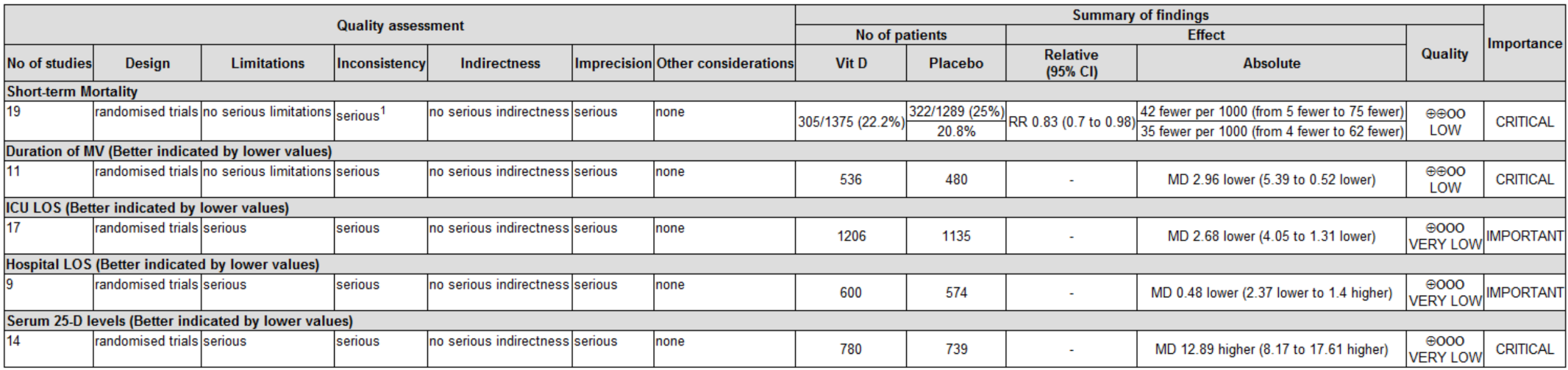


**Additional File 7**

**Funnel plot of comparison: Short-term mortality**


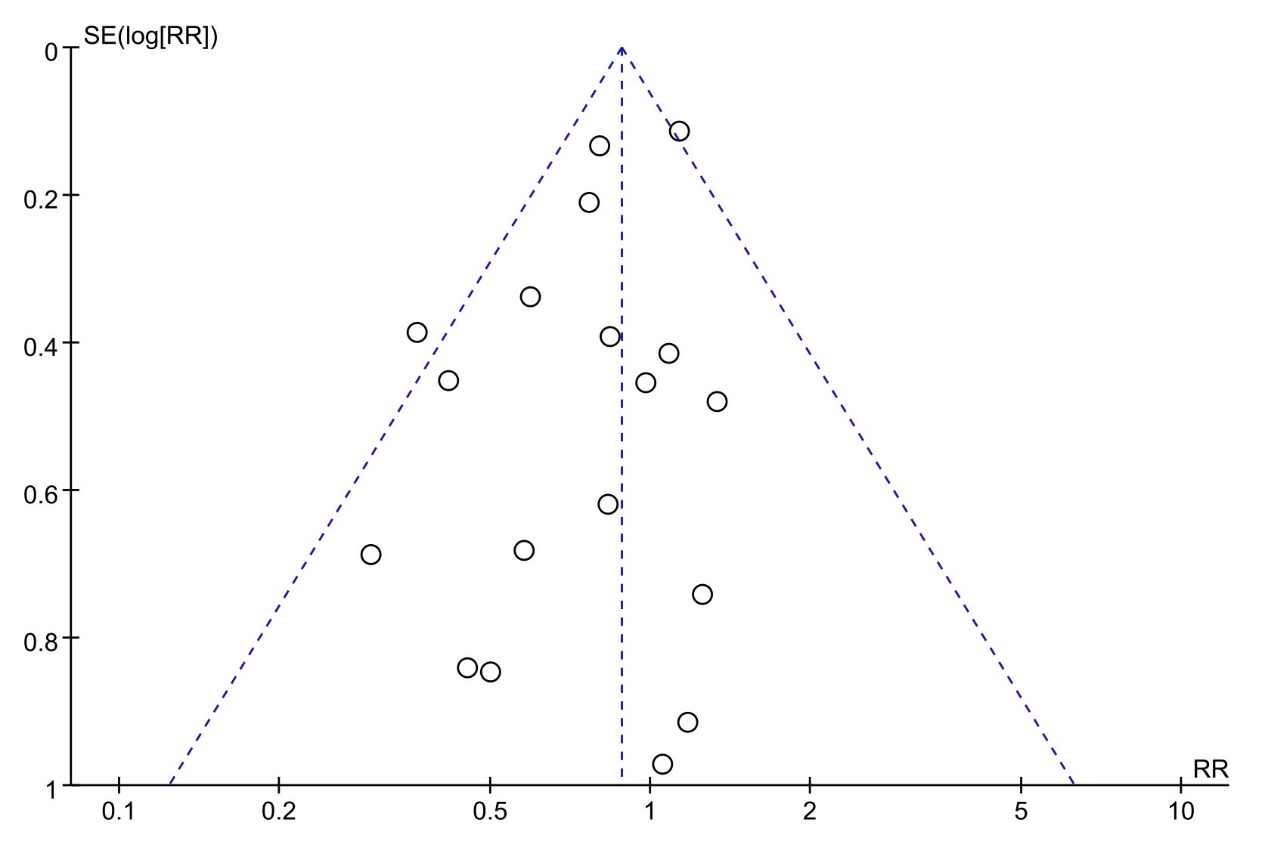


**Additional File 8:**

**Serum 25-hydroxyvitamin D level at baseline and after treatment (ng/ml) and their changes in levels after treatment between the vit D and control groups.**

| Study | Vitamin regimen | Vit D group  (baseline) | Vit D group  (after treatment) | Changes in levels (Vit D group) | Days after treatment | Control group  (baseline) | Control group (after treatment) | Changes in levels (Control group) |
| --- | --- | --- | --- | --- | --- | --- | --- | --- |
| Amrein 2011 | H/EN | 13.1±2.0 | 38.2±16.5 | **25.1±15.6** | 7 | 14.1±3.7 | 13.7±4.1 | **-0.4±4** |
| Amrein 2014 | H/EN | 13±4 | 35.5±20.6 | **22.5±18.9** | 7 | 13.1±4.3 | 14.5±5.1 | **1.4±4.8** |
| Bhattacharyya 2021 | H/EN | 12.05±6.19 | 17.73±7.57 | **5.7±7** | 5 | 15.47±10.58 | 16.4±13.47 | **0.9±12.3** |
| Ginde 2019 | H/EN | 11.2±4.8 | 46.9±23.2 | **35.7±21.2** | 3 | 11±4.7 | 11.4±5.6 | **0.4±5.2** |
| Han 2016 | H/EN  L/EN | 23.2±7.8^a^  20±7.3^b^ | 45±20  55±14 | **21.8±17.5**  **35±12.1** | 7 | 21.5±12.2 |  | **0.4±7** |
| Hasanloei 2019 | H/EM  L /IM | 17.09±4.53^c^  18.66±3.28^d^ | 28.6±4.02  29.43±5.18 | **11.6±4.3**  **10.8±4.5** | 7 | 17±3.25 | 16±2.71 | **-1±3** |
| Karsy 2019 | H/EN | 14.6±4.2 | 20.8±9.3 | **6.2±8.1** | 3 | 13.9±4.6 | 12.8±4.8 | **-1.1±4.7** |
| Miri 2019 | L/IM | 8.4±6.8 | 10.5±9.8 | **2.1±8.7** | 7 | 11.3±18.23 | 11.2±18.22 | **-0.1±18.2** |
| Naguib 2020 | EN | 21±11.2 | +25.2% | **+25.2%** | 4 | 19.1±9.5 | -9.2% | **-9.2%** |
| Parekh 2018 | L/EN | 47.4 (32-67.8) | 54.9 (43.3-69.4) | **7.5±23.6** | 3 | 46.3 (35.4-69) | 28 (19.4-40.4) | **-7.3±8.7** |
| Quraishi 2015 | L/EN  H/EN | 15 (12-20)^e^  17 (13-25)^f^ | 22 (16-25)  29 (23-41) | **7±6.3**  **12±11.7** | 5 | 19 (13-22) | 19 (11-23) | **-0±8** |
| Sharma 2021 | L/EN | 18.3 (14.5-22.95) | 39.1 (36.75-44.58) | **20.8±6** | 14 | 15.1 (11.8-26.9) | 27.3 (14.6-30.8) | **12.2±11.7** |
| Sistanizad 2021 | L/IM | 7.2±2.31 | 11.1±3.70 | **3.9±3.2** | 7 | 5.2±0.71 | 4.8±0.97 | **-0.4±0.9** |
| Ingels 2020 | IV | 9.2±4.4 | 16±2.1 | **6.8±3.8** | 7 | 8.1±3.8 | 9.2±1.3 | **1.1±3.3** |
| Wang 2024 | IV/EN | 14.4 (11.5–17.1) | 28.5 (20.2–52.6) | **14.1±22.1** | 7 | 13.1 (11.0–16.8) | 13.9 (11.6–18.8) | **0.8±4.9** |

Data were presented as mean±standard deviation or median (Interquartile range). a=250000 UI, b=500000 UI, c=enteral vitamin D, d=intravenous vitamin D, e=200000UI, f=400000UI.

EN=enteral; H=high dose group; IM=intramuscular injection; IV=intravenous; L=low dose group, Vit D=vitamin D.

**Additional File 9:**

**Changes in Serum 25-hydroxyvitamin D level (Δ25[OH]D) after treatment (ng/ml) in the four subgroup patients**

| Study | N | High dose^a^ | Low dose^b^ | IM/IV^c^ | EN^d^ |
| --- | --- | --- | --- | --- | --- |
| Amrein 2011 | 12 | 25.1±15.6 |  |  | 25.1±15.6 |
| Amrein 2014 | 237 | 22.5±18.9 |  |  | 22.5±18.9 |
| Bhattacharyya 2021 | 63 | 5.7±7 |  |  | 5.7±7 |
| Ginde 2019 | 531 | 35.7±21.2 |  |  | 35.7±21.2 |
| Han 2016 | 11  9 | 35±12.1 | 21.8±17.5 |  | 21.8±17.5  35±12.1 |
| Hasanloei 2019 | 24  24 | 11.6±4.3 | 10.8±4.5 | 10.8±4.5 | 11.6±4.3 |
| Karsy 2019 | 134 | 6.2±8.1 |  |  | 6.2±8.1 |
| Miri 2019 | 40 |  | 2.1±8.7 | 2.1±8.7 |  |
| Parekh 2018 | 79 |  | 7.5±23.6 |  | 7.5±23.6 |
| Quraishi 2015 | 10  10 | 12±11.7 | 7±6.3 |  | 7±6.3  12±11.7 |
| Sharma 2021 | 35 |  | 20.8±6 |  | 20.8±6 |
| Sistanizad 2021 | 36 |  | 3.9±3.2 | 3.9±3.2 |  |
| Wang 2014 | 61 | 14.1±22.1 |  |  | 14.1±22.1 |
| Mean difference |  | 18.962 | 9.964 | 5.724 | 17.157 |
| 95%CI |  | 9.859-28.065 | 4.721-15.206 | 0.386-11.062 | 9.643-24.671 |

a>300000 UI, b≤300000 UI, c=intravenous or intramuscular injection, d=enteral vitamin D.

EN=enteral; IM=intramuscular; IV=intravenous.

**Additional File 10:**

**Meta-regression analyses in outcome of mortality among included studies based on several clinical factors:**

A: mv=the proportion of patients with MV (100% group vs. partly group);

B: route=route of vitamin D administration (enteral/oral vs. intravenous/intramuscular injection);

C: dose=vitamin D dose (≤300000 UI vs. >300000 UI);

D: vdd=serum vitamin D level at baseline (VDD, defined as <30 ng/ml vs. no threshold);

E: mc=design (single vs. multicenter study).

**A**


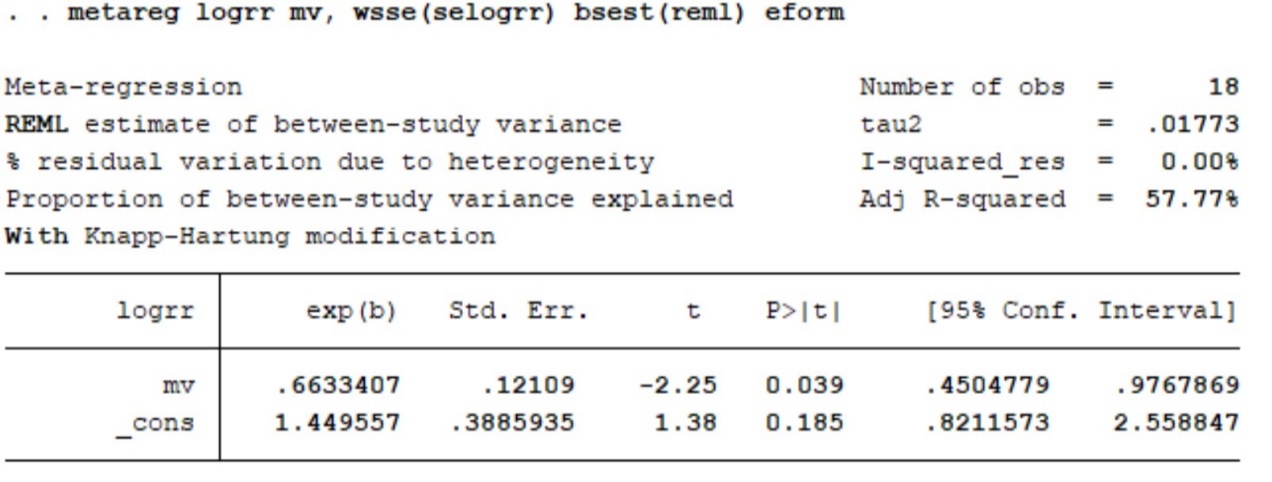


**B**


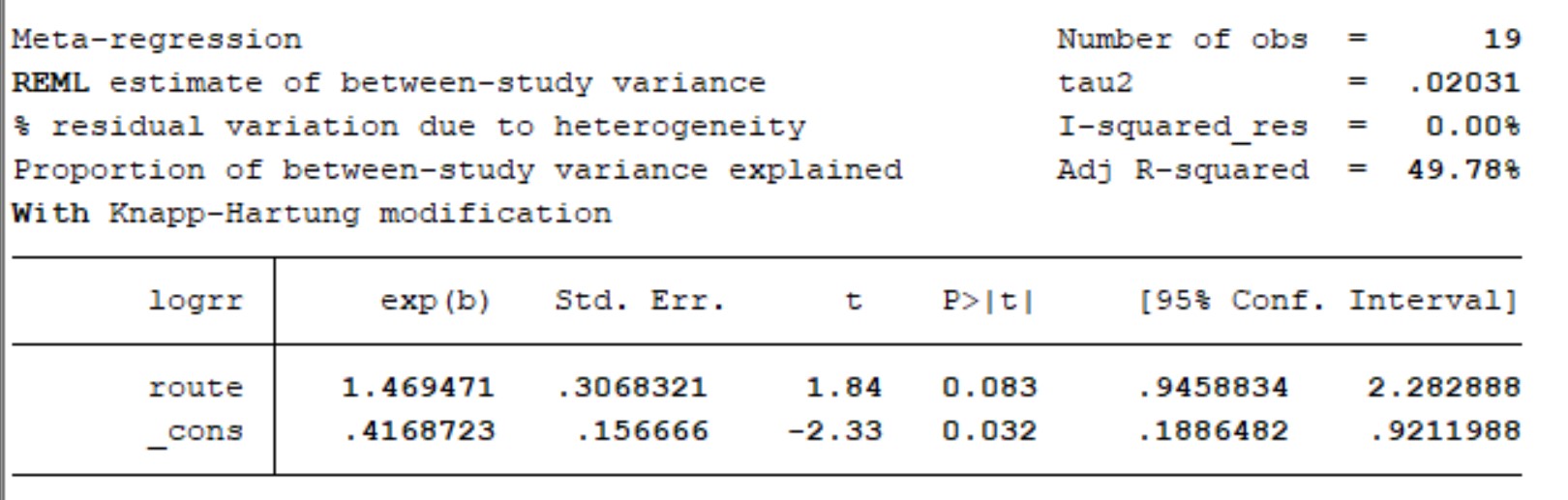


**C**


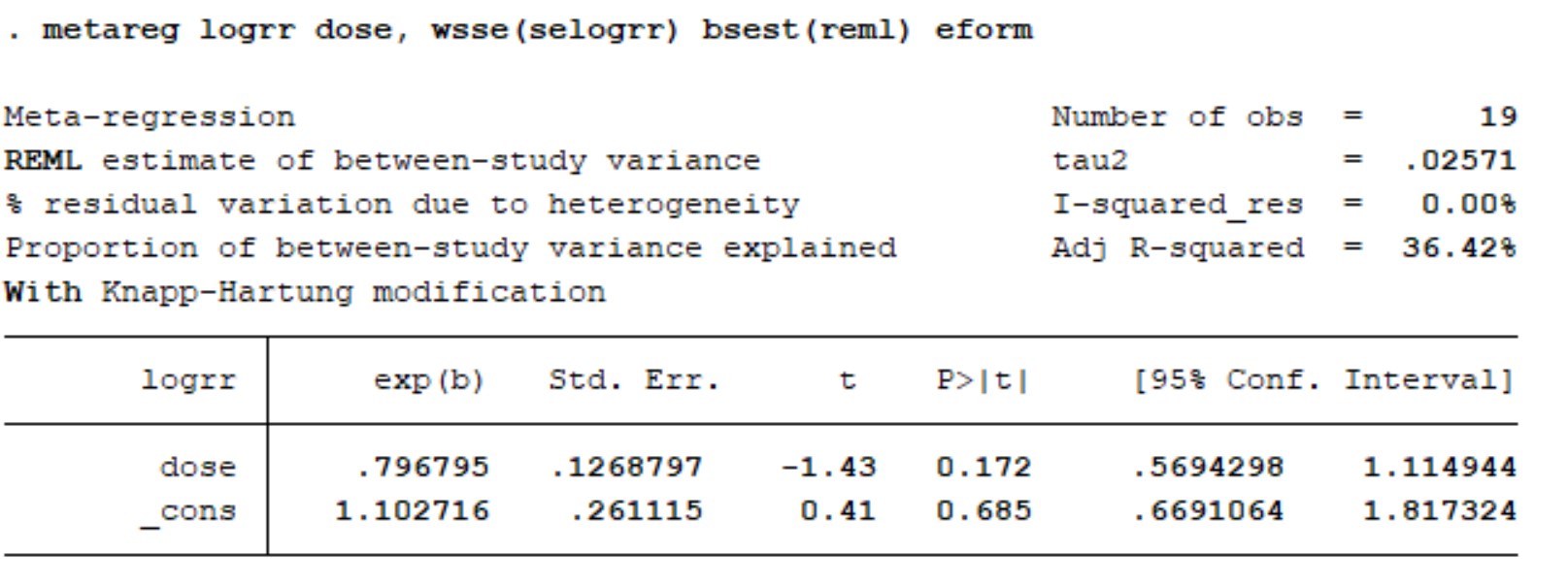


**D**


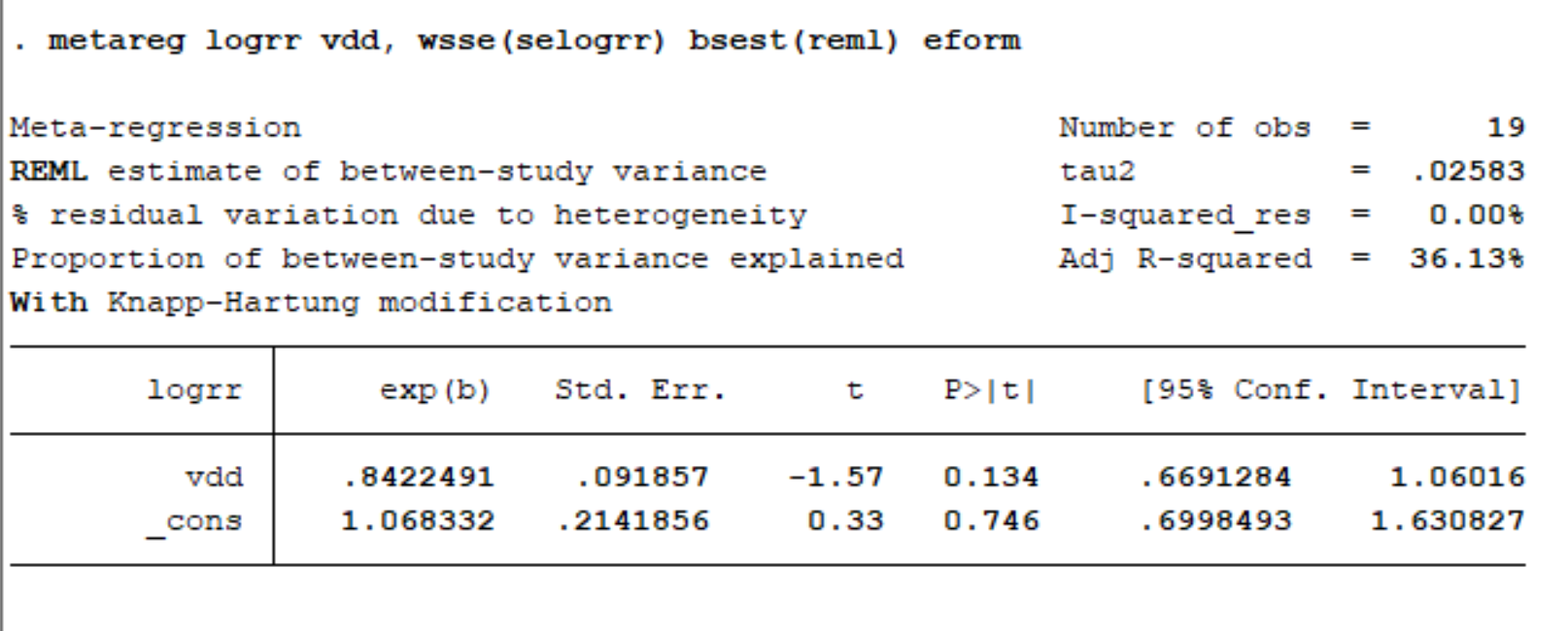


**E**


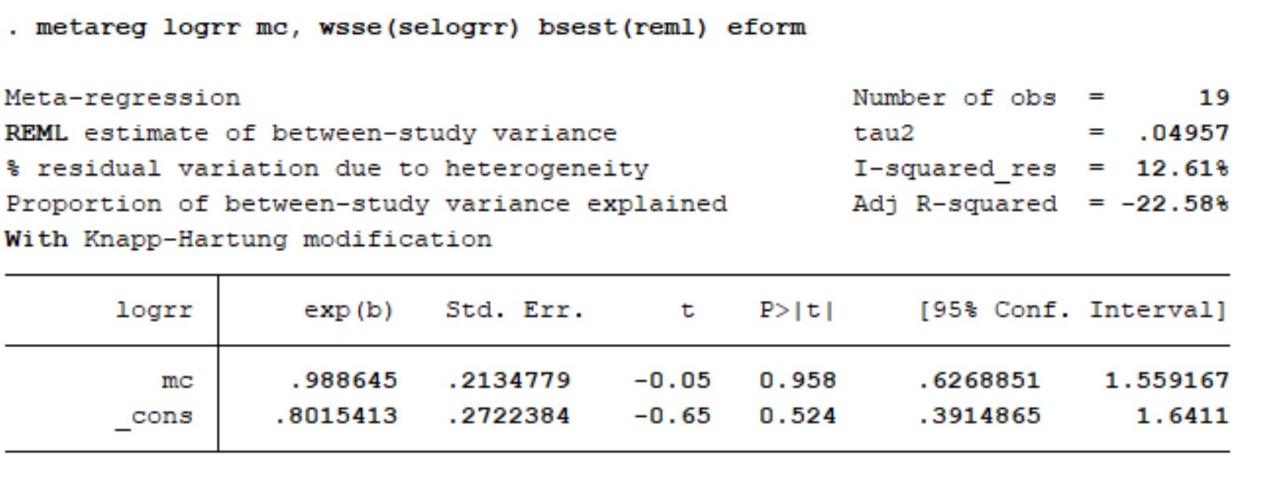


**Additional File 10: Trial sequential analysis of short-term mortality**


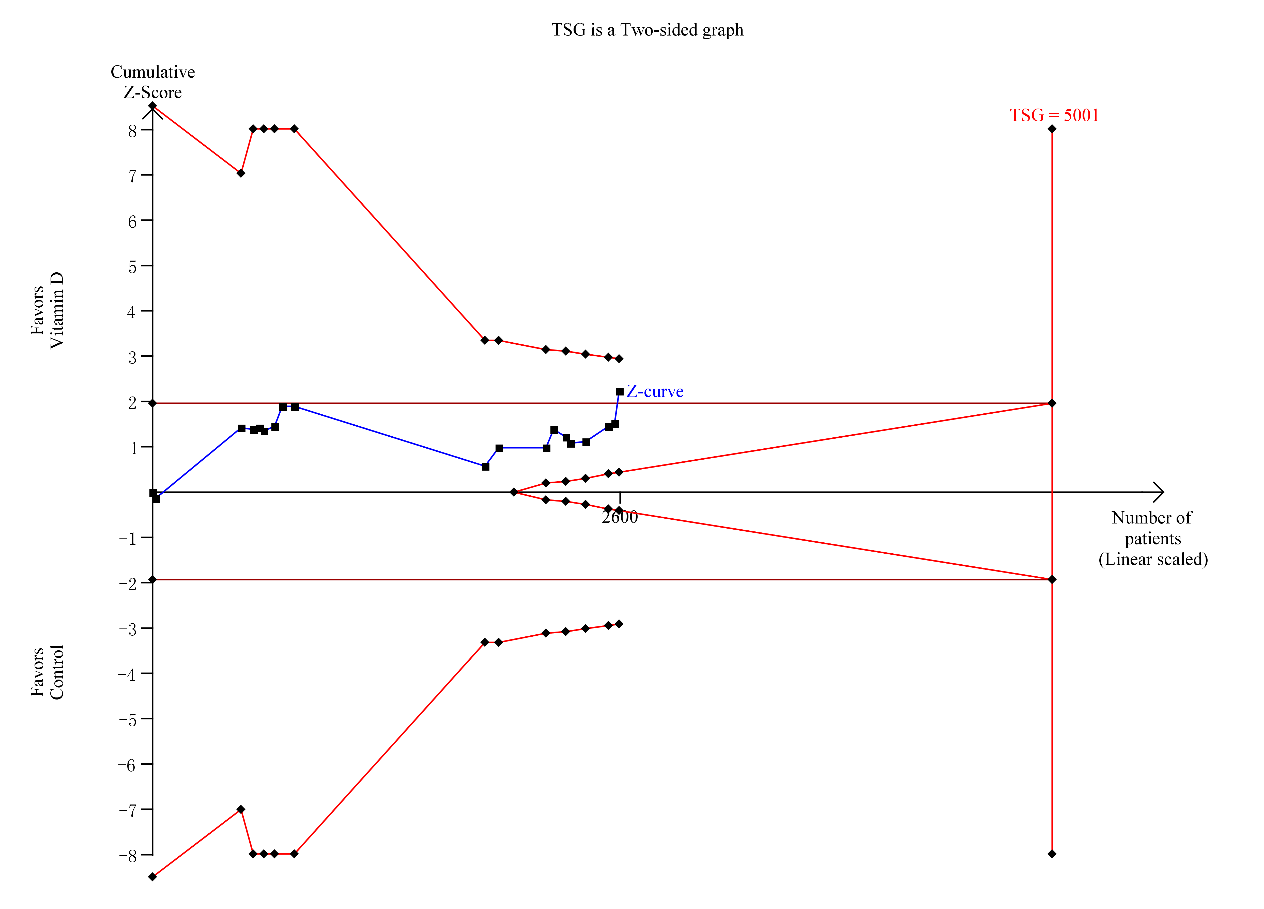


We defined the required information size for decreased mortality based on a risk of type I error of 5%, a risk of type 2 error of 20%, the control group outcome, and a relative risk reduction of 24.8% and 20% to calculate the required information size and the cumulative Z-curve’s eventual breach of relevant trial sequential monitoring boundaries.
